# Analysis of the status and influencing factors of early ambulation in patients with pelvic floor dysfunction after surgery

**DOI:** 10.3389/fmed.2026.1849472

**Published:** 2026-07-15

**Authors:** Chen Qin, Luo Dongmei, Huang Longxian, Xu Min

**Affiliations:** 1 Department of Nursing, The First Affiliated Hospital of Chongqing Medical University, Chongqing, China; 2 Department of Nursing, Sichuan Clinical Research Center for Cancer, Sichuan Cancer Hospital and Institute, Sichuan Cancer Center, Affiliated Cancer Hospital of University of Electronic Science and Technology of China, Chengdu, China; 3 Department of Nephrology II, The First Affiliated Hospital of Kunming Medical University, Kunming, Yunnan, China

**Keywords:** early ambulation, Enhanced Recovery After Surgery, pelvic floor dysfunction, pelvic floor reconstruction, postoperative kinesiophobia

## Abstract

**Objective:**

To investigate the status of early ambulation (within24 h) among patients undergoing surgery for pelvic floor disorders (PFDs) and to identify its independent influencing factors aims to provide evidence-based support for establishing an enhanced recovery (ERAS) nursing pathway for gynecological pelvic floor surgery, and Promote postoperative recovery of PFDs patients and improve their quality of life

**Methods:**

A cross-sectional study was conducted, enrolling 161 patients with PFDs who underwent pelvic floor reconstructive surgery at multiple tertiary hospitals in Chongqing from December 2024 to April 2025. According to the International ERAS Society guidelines for gynecological surgery, early ambulation was defined as “patients independently or with assistance from medical staff and auxiliary devices performing out-of-bed activities within 24 h postoperatively, including: sitting in a chair for ≥20 min after bed-to-chair transfer, and/or standing beside the bed for ≥3 min, and/or walking distance ≥5 meters”. Data were collected through questionnaire surveys, including general patient information, surgical information, self-efficacy, and kinesiophobia scores. Univariate and multivariate Logistic regression analyses were employed to identify factors influencing early ambulation.

**Results:**

Among the 161 patients, 39% (*n* = 63) successfully completed early ambulation within 24 h post-surgery. Multivariate logistic regression analysis revealed that the presence of vaginal packing postoperatively (OR = 3.8, 95%CI:1.6∼9.0, *P* = 0.003); low patient willingness to be active active (OR = 12.3,95%CI:3.6∼41.7, *P* < 0.001); lack of guidance on early ambulation (OR = 4.9,95%CI:1.1∼21.5, *P* = 0.036) were significant risk factors for the failure to ambulate early. Additionally, the incidence of postoperative kinesiophobia among PFDs patients was 84% (135/161), with a TSK-11 score of (29.9 ± 3.1) points, suggesting that kinesiophobia may be a psychological barrier to early ambulation.

**Conclusion:**

The completion rate of ambulation within 24 h after surgery for PFD patients is relatively low postoperative vaginal packing gauze; insufficient willingness to be active; lack of early ambulation guidance, and postoperative kinesiophobia are the main barriers. Clinical medical staff should seek the best evidence, optimize the ERAS strategy, scientifically manage the vaginal packing strategy, strengthen health education for patients and caregivers during the perioperative period, promptly guide patients’ phobia, implement individualized early ambulation programs, improve the compliance of early ambulation activities, and thereby accelerate the postoperative recovery of patients.

## Introduction

Pelvic floor disorders (PFDs) include pelvic organ prolapse (POP), urinary incontinence (UI) and fecal incontinence (FI) are prevalent medical conditions (1), The weighted prevalence of at least one pelvic floor disorder was 23.7% (2), constituting a significant public health issue that affects the lives of millions of adult women worldwide (3). Surgical treatment stands a core component within the therapeutic strategies for PFDs (4), Postoperative rehabilitation is a critical component for enhancing surgical outcomes and reducing complications. Early ambulation, as a central element of postoperative rehabilitation, directly influences patient prognosis (5). Under the guidance of Enhanced Recovery After Surgery (ERAS) protocols, studies have confirmed that early ambulation after pelvic floor reconstruction surgery (PFRS) can effectively reduce bed-related complications such as atelectasis and venous thrombosis, shorten hospital stay, and reduce healthcare costs, thereby providing significant recovery benefits for patients (6–8). Multiple expert consensus statements recommend that patients should ambulate within 24 h after surgery (9–12). However, existing studies have shown that despite the adoption of ERAS, the time to first ambulation time has indeed been advanced compared to traditional models, but it still falls short of the ideal target (6, 7).

Moreover, current literature primarily focuses on the overall implementation of ERAS, with insufficient attention given to the specific issue of “why PFDs patients fail to ambulate early.” In light of this, this study aims to clarify the current status of postoperative ambulation within 24 h for PFDs patients through a cross-sectional survey and systematically analyze its influencing factors. This study is intended to inform the development of precise and operable early ambulation rehabilitation strategies in clinical practice, thereby promoting postoperative recovery and enhancing patients’ quality of life.

## Materials and methods study cohort

Newly diagnosed PFDs patients (aged ≥ 18 years) who underwent PFRS were prospectively enrolled between December 2024 and April 2025 across three tertiary hospitals in Chongqing China. The exclusion criteria included patients who (a) had language, hearing, or cognitive impairments; (b) presented with concurrent lower limb fractures or neurological disorders affecting mobility; and (c) were unable to meet safety criteria for ambulation within 24 h postoperatively. PFDs patients received a standardized, self-completed questionnaire, and detailed clinical indicators were collected after the first time of ambulation. Only one staff member was responsible for collecting questionnaire answers and matching clinical information, and the patients’ personal information was anonymized in the subsequent analysis of the data. Written informed consent was obtained from all participants. The procedures followed in this study were approved by the Ethics Committee of the participating hospitals (approval no. 2024-285-01). The sample size was estimated based on the rule of thumb requiring 5–10 participants per independent variable. This guideline has been widely recommended in multivariate analysis literature (13). With 23 independent variables, the preliminary estimate ranged from 115 to 230 participants. Considering a 10% invalid questionnaire rate, the final sample size range was set at 126–253, with an actual sample size of 161. Completed questionnaires were collected on-site and checked for errors, which were corrected immediately upon discovery.

Data entry was double-checked for accuracy. A total of 161 questionnaires were distributed and collected, with 161 valid responses, achieving a 100% effective response rate.

### Definition of early ambulation

Currently, there is no unified definition for the timing of “early ambulation” after PFRS. Based on relevant international guidelines and consensus statements (9–12, 14, 15), this study defines early ambulation as the first ambulation performed within 24 h postoperatively, either independently or with assistance from medical staff and/or assistive devices. This includes: sitting on the edge of the bed or chair for at least 20 min, standing at the bedside for at least 3 min, and/or walking a distance of at least 5 meters.

### Assessment of kinesiophobia

The Tampa Scale of Kinesiophobia-11 (TSK-11), as revised by Woby et al. (16), is an 11-item questionnaire designed to assess fear of movement. It employs a 4-point Likert scale for each item, resulting in a total score ranging from 11 to 44. Scores exceeding 17 are indicative of kinesiophobia. The scale demonstrates strong psychometric properties, with a Cronbach’s α of 0.79 and a test-retest reliability coefficient of 0.81 (17).

### Assessment of self-efficacy

General self-efficacy scale (GSES) was developed by Schwarzer et al. (18), which is a widely used instrument for assessing an individual’s belief in their capability to achieve goals across various situations. This 10-item scale utilizes a 4-point Likert scoring system, with the total score ranging from 10 to 40; higher scores indicate greater self-efficacy. The scale has a Cronbach’s α of 0.87.

### Data analysis

Statistical analysis was performed using SPSS version 26.0 (IBM Corp., Armonk, NY, USA). Continuous variables were tested for normality using the Shapiro-Wilk test; normally distributed variables were expressed as mean ± standard deviation and compared between groups using the independent samples *t*-test, while non-normally distributed variables were presented as median (interquartile range [P25–P75]) and compared using the Mann-Whitney U test. Categorical variables were reported as frequencies (percentages) and analyzed using the chi-square test or Fisher’s exact test, as appropriate. Binary logistic regression was used to identify factors influencing early ambulation after pelvic floor reconstruction surgery. All hypothesis tests were two-tailed, and a *P*-value < 0.05 was considered statistically significant.

## Results

A total of 161 PFDs patients were recruited, whose clinical information is shown in Table 1. Their ages ranging from 27 to 83 years, patients aged ≥60 years accounted for 55.9%; The mean body mass index (BMI) was 24.17 ± 2.91, at the upper limit of the normal weight range, suggesting an overall higher weight level among participants, This finding aligns with previous literature indicating that women over the age of 50 are more likely to have PFD than younger women and a higher BMI increases the risk and severity of PFDs (2, 19).

**TABLE 1:** General characteristics and univariate analysis results of included PFDs patients.

Variables	Total (*n* = 161)	Early ambulation group (*n* = 98)	Non-early ambulation group (*n* = 63)	Statistic	*P*
Age(y), *n* (%)				χ^2^ = 4.1	**0.043**
	<60	71 (44.1)	37 (37.8)	34 (54.0)	
	= 60	90 (56.0)	61 (62.2)	29 (46.0)	
BMI (kg/m^2^), *n* (%)				–	0.581
	<18.5	1 (0.6)	1 (1.0)	0 (0.0)	
	18.5–23.9	75 (46.6)	44 (44.9)	31 (49.2)	
	24–27.9	69 (42.9)	41 (41.8)	28 (44.4)	
	= 28	16 (9.9)	12 (12.2)	4 (6.4)	
Education background, *n* (%)			–	0.189	
Primary school and below	75 (46.6)	50 (51.0)	25 (39.7)		
Junior high school	48 (29.8)	28 (28.6)	20 (31.8)		
High school and technical secondary school	25 (15.5)	16 (16.3)	9 (14.3)		
Junior college	7 (4.4)	2 (2.0)	5 (7.9)		
Bachelor’s degree or above	6 (3.7)	2 (2.0)	4 (6.4)		
Marital status, *n* (%)				–	0.365
Single / unmarried	0 (0.0)	0 (0.0)	0 (0.0)		
Married	145 (90.1)	86 (87.8)	59 (93.7)		
Divorced	2 (1.2)	1 (1.0)	1 (1.6)		
Widowed	14 (8.7)	11 (11.2)	3 (4.8)		
Residence, *n* (%)				χ^2^ = 4.5	0.107
Urban	102 (63.4)	58 (59.2)	44 (69.8)		
Town / Small Town	37 (23.0)	28 (28.6)	9 (14.3)		
Rural	22 (13.7)	12 (12.2)	10 (15.9)		
Payment method, *n* (%)				–	0.395
Medical insurance	154 (95.7)	95 (96.9)	59 (93.7)		
Commercial insurance	1 (0.6)	0 (0.0)	1 (1.6)		
Self-pay	6 (3.7)	3 (3.1)	3 (4.8)		
Willingness to ambulate within 24 h, *n* (%)				χ^2^ = 38.8	<**0.001**
Unwilling	85 (52.8)	69 (70.4)	16 (25.4)		
Willing	76 (47.2)	29 (29.6)	47 (74.6)		
Caregiver belief about early ambulation, *n* (%)				–	<**0.001**
Bed rest	87 (54.0)	69 (70.4)	18 (28.6)		
Early ambulation	66 (41.0)	23 (23.5)	43 (68.3)		
Other	8 (5.0)	6 (6.1)	2 (3.2)		
Received guidance for early ambulation, *n* (%)				χ^2^ = 14.8	<**0.001**
No	32 (19.9)	29 (29.6)	3 (4.8)		
Yes	129 (80.1)	69 (70.4)	60 (95.2)		
Pain assessment, *n* (%)				–	0.355
Mild	91 (56.5)	58 (59.2)	33 (52.4)		
Moderate	69 (42.9)	40 (40.8)	29 (46.0)		
Severe	1 (0.6)	0 (0.0)	1 (1.6)		
POP staging, *n* (%)				–	0.050
I	14 (8.7)	11 (11.2)	3 (4.8)		
II	72 (44.7)	44 (44.9)	28 (44.4)		
III	71 (44.1)	43 (43.9)	28 (44.4)		
IV	4 (2.5)	0 (0.0)	4 (6.4)		
Number of comorbidities, *n* (%) None	35 (21.7)	14 (14.3)	21 (33.3)	χ^2^ = 10.3	**0.006**
1—3	60 (37.3)	36 (36.7)	24 (38.0)		
≥3	66 (41.0)	48 (49.0)	18 (28.6)		
Disease duration, *n* (%)				χ^2^ = 2.5	0.283
>24 months	62 (38.51)	33 (33.67)	29 (46.03)		
6–24 months	45 (28.0)	29 (29.6)	16 (25.4)		
≤6 months	54 (33.5)	36 (36.7)	18 (28.6)		
Surgical time, *n* (%) ≤90 min	90 (55.9)	52 (53.1)	38 (60.3)	χ^2^ = 0.8	0.365
>90 min	71 (44.1)	46 (46.9)	25 (39.7)		
ASA classification, *n* (%)				χ^2^ = 3.0	0.085
II	110 (68.3)	62 (63.3)	48 (76.2)		
III	51 (31.7)	36 (36.7)	15 (23.8)		
Surgical approach, *n* (%)				χ^2^ = 4.4	0.112
Laparoscopic	40 (24.8)	19 (19.4)	21 (33.3)		
Vaginal	76 (47.2)	48 (49.0)	28 (44.4)		
Combined laparoscopic + vaginal	45 (28.0)	31 (31.6)	14 (22.2)		
Hysterectomy performed, *n* (%)				χ^2^ = 4.6	**0.032**
No	115 (71.4)	64 (65.3)	51 (81.0)		
Yes	46 (28.6)	34 (34.7)	12 (19.1)		
Vaginal gauze removal time, *n* (%) ≥24 h	111 (68.9)	85 (86.73	26 (41.3)	χ^2^ = 41.7	<**0.001**
≤24 h	36 (22.4)	6 (6.1)	30 (47.6)		
Not packed	14 (8.7)	7 (7.1)	7 (11.1)		
Postoperative hemoglobin level, g/L, *n* (%)				–	**0.031**
>110	97 (60.3)	65 (66.3)	32 (50.8)		
90–110	62 (38.5)	33 (33.7)	29 (46.0)		
<90	2 (1.2)	0 (0.0)	2 (3.2)		
Pre-postoperative hemoglobin difference, *n* (%) ≤5	12 (7.5)	11 (11.2)	1 (1.6)	χ^2^ = 6.9	**0.032**
5–10	21 (13.0)	15 (15.3)	6 (9.5)		
≥10	128 (79.5)	72 (73.5)	56 (88.9)		
Catheter indwelling time, *n* (%) ≤24 h	151 (93.8)	98 (100.0)	53 (84.1)	χ^2^ = 14.0	<**0.001**
>24 h	10 (6.2)	0 (0.0)	10 (15.8)		
TSK score, M (Q1, Q3)	29.0 (27.0, 33.0)	29.0 (27.0, 33.8)	29.0 (26.0, 33.0)	*Z* = −0.5	0.6
GCES score, M (Q1, Q3)	24.0 (20.0, 30.0)	22.50 (20.0, 30.0)	30.0 (20.0, 30.0)	*Z* = −1.3	0.2
Postoperative fatigue score, M (Q1, Q3)	2.0 (0.0, 4.0)	2.00 (0.0, 4.0)	2.0 (0.0, 4.0)	*Z* = −0.1	0.9

“−” denotes Fisher’s exact test; TSK-11, Tampa Scale of Kinesiophobia-11; GSES, General self-efficacy scale. Bold values represent variables that showed statistically significant differences in univariate analysis.

As showed in Table 1, Only 39.1% (63 cases) of patients completed early ambulation within 24 h postoperatively. Based on early ambulation status, patients were divided into the Early ambulation group and the Non-early ambulation group. Univariate analysis revealed statistically significant differences (*P* < 0.05) between the two groups in the following variables: age; willingness to ambulate within 24 h; caregiver belief about early ambulation; received guidance for early ambulation; Number of comorbidities; hysterectomy performed; Vaginal gauze removal time; postoperative hemoglobin level; pre-postoperative hemoglobin difference, catheter indwelling time. Variables that were significant (*P* < 0.05) in the univariate analysis were subsequently included in the logistic regression analysis.

### Multivariate logistic regression results

Based on the univariate analysis results (*P* < 0.05), variables identified as statistically significant were entered as independent variables, with postoperative ambulation within 24 h as the dependent variable, to conduct a stepwise logistic regression analysis. The analysis revealed that three variables—time to vaginal gauze removal, patients’ willingness to ambulate within 24 h, and received guidance for early ambulation—entered the regression model, all serving as independent predictors of early postoperative ambulation (*P* < 0.05). Specifically, early removal of vaginal gauze was identified as an independent facilitating factor for ambulation within 24 h postoperatively. Patients who had the gauze removed within 24 h were 12.6 times more likely to ambulate within the same period compared to those whose gauze was removed after 24 h. Moreover, the willingness to ambulate within 24 h postoperatively independently facilitated early ambulation, with willing patients being 7.8 times more likely to ambulate within 24 h than those who were unwilling. Additionally, patients who received early ambulation guidance had a 4.9-fold higher probability of ambulating within 24 h compared to those who did not receive such guidance. Detailed results are presented in Tables 2.

**TABLE 2:** Logistic stepwise regression analysis results of included PFDs patients (*n* = 161).

Variables	β	S.E	Wald	*P*	OR (95%CI)
Age(y)					
<60					1.0 (Reference)
≥60	−0.6	0.5	1.0	0.3	0.6 (0.2 ∼ 1.7)
Number of comorbidities					
None					1.0 (Reference)
1—3	−0.3	0.7	0.3	0.6	0.7 (0.2 ∼ 2.7)
≥3	−0.5	0.7	0.6	0.4	0.6 (0.2 ∼ 2.3)
Hysterectomy performed					
No					1.0 (Reference)
Yes	−0.4	0.6	0.6	0.5	0.6 (0.2 ∼ 2.1)
Vaginal gauze removal time					
≥24 h					1.0 (Reference)
≤24 h	2.5	0.8	11.0	<0	12.6 (2.8 ∼ 56.2)
Not packed	1.2	0.8	2.1	0.2	3.2 (0.7 ∼ 15.5)
Postoperative hemoglobin level, g/L					
>110					1.0 (Reference)
90–110	−0.3	0.5	0.3	0.6	0.7 (0.3 ∼ 2.0)
<90	13.6	4029.7	0.0	1.0	842329.9 (0.0 ∼ Inf)
Pre-postoperative hemoglobin difference, g/L					
≤5					1.0 (Reference)
5–10	1.7	1.3	1.7	0.2	5.6 (0.4 ∼ 77.5)
≥10	1.8	1.2	2.1	0.1	5.943 (0.5 ∼ 66.8)
Catheter indwelling time					
≤24					1.0 (Reference)
>24	18.0	1733.3	0.0	1.0	64358071.6(0.0∼ Inf)
Willingness to ambulate within 24 h					
Unwilling					1.0(Reference)
Willing	2.1	0.7	9.2	0.00	7.8 (2.1 ∼ 29.3)
Caregiver belief about early ambulation					
Bed rest					1.0 (Reference)
Early ambulation	1.1	0.7	2.9	0.1	3.0(0.9 ∼ 10.3)
Other	−0.4	1.3	0.1	0.8	0.665 (0.1 ∼ 8.6)
Received guidance for early ambulation					
No					1.0 (Reference)
Yes	1.6	0.8	4.4	0.0	4.9(1.1 ∼ 21.5)

OR, odds ratio; CI, confidence interval.

### Postoperative kinesiophobia scores

This study also revealed the kinesiophobia scores associated with early postoperative ambulation among included PFDs patients. The postoperative kinesiophobia score for these patients was 29.9 points, with 84% of patients experiencing kinesiophobia. A comparative analysis between the kinesiophobia group and the non-kinesiophobia group showed a statistically significant difference in the total TSK-11 score (*P* < 0.05). Notably, both the activity avoidance dimension and the somatic focus dimension exhibited statistically significant differences (*P* < 0.05). The kinesiophobia group scored higher in the total score and in both dimensions compared to the non-kinesiophobia group, with the activity avoidance dimension scoring the highest. This suggests that patients primarily fear the adverse consequences associated with activity. Detailed results are presented in Table 3.

**TABLE 3:** Analysis of the differences between the occurrence status of postoperative kinesiophobia and the TSK score dimension in patients after pelvic floor reconstruction surgery (*n* = 161).

Variables	Non-kinesiophobia group (*n* = 25)	Kinesiophobia group (*n* = 136)	t/χ^2^	*P*
TSK-11 total score, Mean ± SD	24.8 ± 1.4	30.8 ± 2.3	*t* = −17.1	<0.001
Activity avoidance, Mean ± SD	14.4 ± 1.8	18.8 ± 1.8	*t* = −10.8	<0.001
Somatic focus, Mean ± SD	10.4 ± 1.3	12.0 ± 1.3	*t* = −5.8	<0.001

TSK-11 stands for the Tampa Scale of Kinesiophobia-11; The Activity Avoidance dimension corresponds to items 1, 2, 3, 5, 6, 9, and 11, reflecting the belief dimension of avoiding activities due to fear of pain or re-injury.; The Somatic Focus dimension corresponds to items 4, 7, 8, and 10, reflecting the belief dimension of focusing on the presence of serious underlying bodily problems.

## Discussion

### Current status of early ambulation

Current guidelines and expert consensus (9–12, 15) consistently recommend early ambulation as an effective strategy to reduce bed- related complications and accelerate postoperative recovery. However, our study revealed that only 39.1% of patients achieved ambulation within the first 24 h post- surgery. This completion rate aligns with the findings of Xiao et al. (6) but is notably lower than the rates reported by Zhou Linlin et al. (20), Importantly, while some studies have successfully advanced the timing of early ambulation to within 24 h, this benchmark remains considerably later than the ambulation timelines reported for other gynecological abdominal procedures. For instance, patients undergoing radical cervical cancer surgery typically ambulate at 6.5 ± 1.6 h post-operatively (21), and those undergoing laparoscopic procedures at 6.4 ± 0.9 h (22). The delayed implementation observed in our cohort may be attributable to the relatively recent adoption of the “ambulation within 24 h” concept specifically for PFRS. Existing evidence supporting this practice is primarily derived from other gynecological and abdominal surgeries, leading to a cautious approach among healthcare providers, Particularly, there is a concern that early ambulation after mesh implantation surgery might trigger mesh-related complications. Moreover, the lack of a clear, operable early ambulation protocol tailored to PFRS further hinders implementation. Evidence suggests that standardized peri-operative ambulation protocols can effectively promote early ambulation and enhance recovery (23). Future efforts should therefore focus on integrating high- quality evidence into clinical practice, addressing the gap between optimal recommendations and real- world implementation, shifting traditional provider attitudes, and establishing structured early mobilization pathways specific to PFRS.

In addition, our study revealed significant variability in personnel responsible for ambulation: 70.2% of patients were assisted by family members, while only 9.9% received direct assistance from medical staff, 19.9% ambulated independently. This family-dominated pattern, though reflecting our institutional caregiving culture, highlights potential concerns in early ambulation implementation, the minimal direct medical staff participation suggests healthcare providers remain hesitant to lead early ambulation activities. This aligns with the aforementioned concerns about potential complications and the absence of standardized protocols. Therefore, future efforts should establish multidisciplinary early ambulation pathways, including standardized family training programs and enhanced nursing oversight mechanisms to optimize ambulation outcomes.

### Influencing factors analysis

#### Patient willingness and caregiver attitudes

Patient willingness within the first 24 h postoperatively serves not only as a proxy for proactive engagement in rehabilitation but also as a robust predictor of both actual ambulation timing and the overall quality of recovery. In our cohort, approximately 54% of patients expressed concerns regarding early ambulation. The predominant anxieties centered on wound dehiscence, recurrence of prolapse, and the perceived inconvenience associated with vaginal packing and indwelling urinary catheters.

Notably, a substantial proportion (69.6%) explicitly feared prolapse recurrence following ambulation, reflecting a cognitive bias wherein patients mistakenly associate ambulation directly with surgical failure. This bias may be exacerbated by discomforts such as urinary leakage or a sensation of “downward pressure” during activities like coughing or laughing, which can precipitate catastrophic thinking and amplify fear of early ambulation (24). Univariate analysis revealed that caregiver attitudes toward 24 h ambulation were significantly associated with patient mobilization (*P* < 0.001). However, this variable did not retain significance in the final multivariate model, likely due to collinearity with patient willingness. Existing literature (25, 26) indicates that caregiver attitudes often exert indirect effects on patient behavior through mediators such as self-efficacy and pain perception. While the direct predictive effect of caregiver attitudes may be attenuated when these mediators are accounted for, the clinical relevance of caregiver support remains substantial. Studies (27, 28) have demonstrated that caregiver presence, active listening, and encouragement can enhance patients’ sense of being valued, thereby fostering ambulation participation in rehabilitation. Collectively, these findings underscore patient willingness as the decisive “switch” that governs actual ambulation timing and recovery outcomes, whereas caregiver attitudes may modulate compliance indirectly through influencing patient cognition and behavior. Consequently, healthcare providers should prioritize targeted health education and individualized guidance to enhance patient readiness for early ambulation, while simultaneously reinforcing caregiver education to create a supportive environment conducive to optimal postoperative recovery.

### Vaginal packing

Conventional postoperative protocols often advocate for routine vaginal packing, predicated on the belief that it mitigates hematoma formation within the vaginal wall and apex, thereby reducing the risk of infection. In this study, 68.9% of patients retained vaginal packing beyond the 24 h postoperative window; 22.4% underwent removal within 24 h, and only 8.7% received no packing at all. It is hypothesized that prolonged vaginal packing may impede early ambulation, as suggested by clinical observations. Patients might experience discomfort during fundamental activities such as turning, sitting, or standing, and harbor concerns about packing displacement.

Nevertheless, this proposed mechanism warrants further investigation through prospective studies designed to specifically evaluate the relationship between vaginal packing and patient comfort during ambulation. Recent expert consensus statements (29) and systematic review (30) explicitly advise against the routine use of postoperative vaginal packing. Moreover, guidelines from the Chinese Medical Association recommend avoiding routine packing except in cases involving transvaginal mesh implantation. Consequently, the role of vaginal packing remains a subject of debate in the gynecologic surgical literature. While some guidelines recommend against its routine use, citing that risks may outweigh benefits for most patients, other studies have reported no increase in postoperative discomfort with potential benefits of reduced bleeding and hematoma formation. Therefore, the decision to pack should be individualized based on the specific surgical procedure, anticipated risk of bleeding, and surgeon experience.

### Postoperative kinesiophobia

Kinesiophobia is a psychosomatic disorder wherein somatic symptoms and functional impairments often trigger a fear of movement. This condition is driven by a complex interplay of physiological, psychological, and social factors, leading patients to excessively worry about exercise, fearing pain exacerbation or injury aggravation (31). Our findings reveal a postoperative kinesiophobia incidence of 84% among patients with PFD, surpassing rates reported in cardiac surgery patients (39.2%–82.6%) (32), cervical spondylosis patients (75.4%) (33), abdominal surgery patients (42.1%) (34), and after pacemaker implantation (60%) (35). This underscores the high prevalence of kinesiophobia in the PFDs cohort. Further analysis indicates that the activity avoidance dimension scored highest in the kinesiophobia group (18.8 ± 1.8), significantly exceeding the non-kinesiophobia group (14.4 ± 1.8). The somatic focus dimension scored relatively lower (12.0 ± 1.3), yet remained significantly higher than the non-kinesiophobia group (10.4 ± 1.3). These findings suggest that postoperative PFDs patients harbor a stronger fear of activity (avoidance behavior) than an overemphasis on bodily symptoms, primarily fearing adverse outcomes from ambulation. This aligns closely with cognitive-behavioral theory (36, 37), wherein PFDs patients develop catastrophic cognitions due to surgical trauma, pain, or fear of prolapse recurrence, leading to erroneous beliefs that “ambulation will cause severe pain or surgical failure.” Concurrently, diminished self-efficacy prompts a “bed rest” strategy, resulting in muscle weakness and orthostatic hypotension, which further “validates” pain presence, establishing a vicious cycle of “fear-avoidance-functional decline.” The high incidence of kinesiophobia in PFD patients may be associated with specific demographic characteristics. This study exclusively enrolled female PFDs patients, a condition predominantly affecting middle-aged and elderly women. Research indicates that women are more sensitive to emotional fluctuations and are more prone to activity avoidance postoperatively (38, 39). Additionally, older adults exhibit higher kinesiophobia score (40). Based on these findings, clinical interventions should shift from merely encouraging ambulation to a cognitive-behavioral model. Early postoperative assessments of kinesiophobia using validated tools should become routine, with particular emphasis on the activity-avoidance dimension to inform comprehensive rehabilitation strategies.

Notably, univariate analysis revealed no significant association between TSK scores and ambulation within 24 h postoperatively (*P* = 0.593), precluding inclusion in the multivariate regression model. This may stem from measurement limitations: the dependent variable “ambulation within 24 h postoperatively” was binary (yes/no), failing to capture activity quality (e.g., distance, steps, Barthel Index).

For PFD patients, “getting out of bed” might represent a highly constrained passive transfer rather than an active, robust functional recovery. Consequently, the impact of kinesiophobia on activity quality was not adequately reflected by a simple “yes/no” metric. Future studies should employ more sensitive functional indicators to accurately capture the true relationship between kinesiophobia and early ambulation.

## Conclusion

In summary, early ambulation (within 24 h) among PFDs patients remains suboptimal. Independent predictors of delayed ambulation include time to vaginal gauze removal, patients’ willingness to ambulate within 24 h, and received guidance for early ambulation. Moreover, postoperative kinesiophobia emerges as a pivotal clinical factor influencing recovery trajectories. To enhance outcomes, clinicians should: Optimize ERAS protocols and vaginal packing management based on the best available evidence; Bolster health education for both patients and caregivers, specifically targeting the mitigation of kinesiophobia; Implement personalized early ambulation plans to improve adherence and accelerate postoperative recovery. While this multi- center cross- sectional study provides valuable insights, its generalizability is limited by sample size and inter- institutional variability. Future large- scale, multi- center prospective investigations are needed to systematically capture postoperative rehabilitation data and furnish higher- quality evidence for individualized activity rehabilitation strategies.

## Data Availability

The original contributions presented in this study are included in this article/supplementary material, further inquiries can be directed to the corresponding author.
